# Behavioural Variant of Frontotemporal Dementia or Mood Disorder?

**DOI:** 10.1192/j.eurpsy.2022.1150

**Published:** 2022-09-01

**Authors:** B. Mesquita, S. Paulino, A. Fraga, J. Facucho-Oliveira, P. Espada-Santos, M. Albuquerque, M. Costa

**Affiliations:** 1 Hospital de Cascais, Psychiatry, Alcabideche, Portugal; 2 Hospital de Cascais, Psychiatry, Coimbra, Portugal

**Keywords:** Dementia, bipolar disorder

## Abstract

**Introduction:**

The behavioural variant of frontotemporal dementia (bvFTD) is a devastating neurodegenerative syndrome with its peak in the early sixties at about 13 per 100,00. The diagnosis of bvFTD relies on clinical assessment as patients present executive and behavioural deficits, like apathy, loss of motivation and personality changes. Current diagnosis criteria lack specificity and symptomatic overlap between bvFTD and primary psychiatric disorders (PPD) pose a diagnostic conundrum, with half of bvFTD patients previously receiving a psychiatric diagnosis.

**Objectives:**

The goal is to discuss the syntomatic overlap of these two entities.

**Methods:**

Brief non-systematic literature review on the topic, illustrated by a case-report presentation.

**Results:**

A 69 year old men, retired and single, is committed for thought and behavior disorganization and insomnia. He presented expansive mood but also temporal and spatial disorientation and periods of incongruous speech. This patient’s clinical presentation could both entice a diagnosis of bvFTD but also of an affective disorder, especially since it has been reported that neuropsychiatric presentations, like late-onset psychosis or mania, can be the initial presentation of this form of dementia, particularly in patients with C9orf72 mutations, who often display persecutory or grandiosity delusions.

**Conclusions:**

This clinical case exemplifies the difficulty that lies in differentiating cases of bvFTD from late-onset idiopathic mood or psychotic disorders. It is important to consider that on cognitive assessment patients with bvFTD score significantly worse on executive function tests that PPD patients No disease- modifying therapies are available for patients with bvFTD, therefore drug treatment should focus on the most disruptive or taggable behaviours.

**Disclosure:**

No significant relationships.

